# Supplementary material for: Population Density and Host Preference of the Japanese Pine Sawyer (Monochamus alternatus) in the Qinling–Daba Mountains of China
Source: Insects. 2023 Feb 13;14(2):181. doi: 10.3390/insects14020181 (PMC9967673; doi:10.3390/insects14020181)
Supplement: Supplementary file 1 [file insects-14-00181-s001.zip › Supplementary Information.pdf]

Supplementary Information

Population Density and Host Preference of the Japanese Pine Sawyer (*Monochamus alternatus*) in the Qinling–Daba Mountains of China

Junke Nan [1,2], Jingyu Qi [2], Yuexiang Yang [2], Mengqin Zhao [2], Chaoqiong Liang [3], Hong He [2,*] and Cong Wei [1,*]

[1] Key Laboratory of Plant Protection Resources and Pest Management of the Ministry of Education, College of Plant Protection, Northwest A&F University, Yangling 712100, China
[2] Key Laboratory of National Forestry and Grassland Administration for Control of Forest Biological Disasters in Western China, College of Forestry, Northwest A&F University, Yangling 712100, China
[3] Shaanxi Academy of Forestry, Xi'an 710082, China

Supplementary figure:

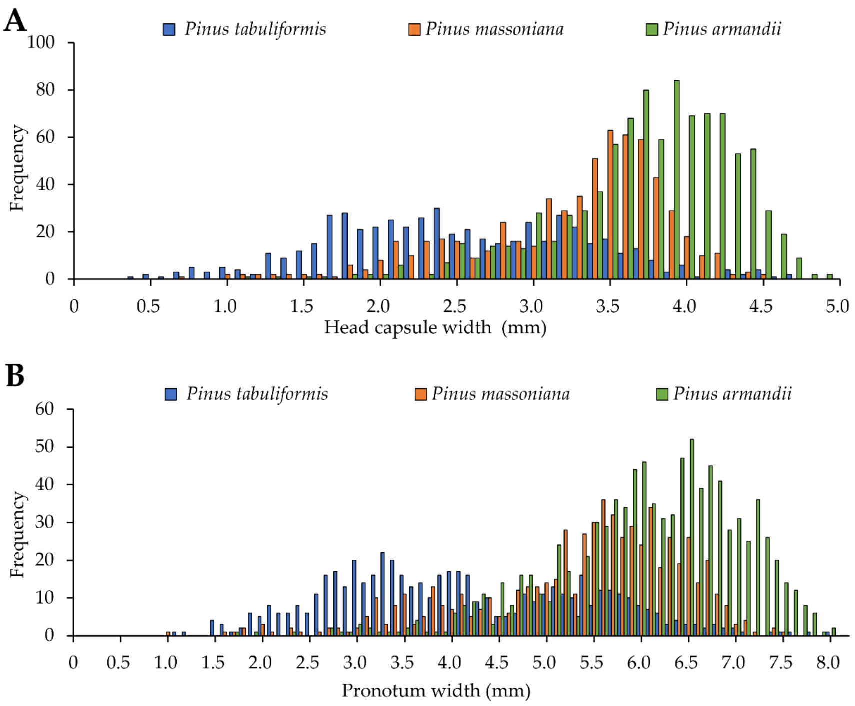

Figure S1. Frequency distribution of head capsule width and pronotum width of *Monochamus alternatus* larvae. A: Frequency distribution of head capsule width of *M. alternatus* larvae on *P. tabuliformis*, *P. armandii* and *P. massoniana*; B: Frequency distribution of pronotum width of *M. alternatus* larvae on *P. tabuliformis*, *P. armandii* and *P. massoniana*.

Supplementary table captions:

Table S1. Different groups were divided according to the head capsule width of *Monochamus alternatus* larvae.

Table S2. Different groups were divided according to the pronotum width of *Monochamus alternatus* larvae.

Table S3. Feeding and oviposition preference of *Monochamus alternatus* on three pine trees.
